# Exploring combat stress exposure effects on burn pain in a female rodent model

**DOI:** 10.1186/s12868-022-00759-z

**Published:** 2022-12-06

**Authors:** Misty M. Strain, Sirima Tongkhuya, Nathan Wienandt, Farah Alsadoon, Roger Chavez, Jamar Daniels, Thomas Garza, Alex V. Trevino, Kenney Wells, Thomas Stark, John Clifford, Natasha M. Sosanya

**Affiliations:** Pain and Sensory Trauma Care, Combat Research Team 5 (CRT5), US Army Institute of Surgical Research (USAISR), JBSA Fort Sam Houston, 3698 Chambers Pass, San Antonio, TX 78234-4504 USA

**Keywords:** Female rodent, Unpredictable combat stressors, Thermal injury, Nociception, Mechanosensitivity

## Abstract

In the military, constant physiological and psychological stress encountered by Soldiers can lead to development of the combat and operational stress reaction (COSR), which can effect pain management. Similar effects are seen in other populations subjected to high levels of stress. Using a model of COSR, our lab recently showed that four weeks of stress prior to an injury increases pain sensitivity in male rats. With the roles of women in the military expanding and recent studies indicating sex differences in stress and pain processing, this study sought to investigate how different amounts of prior stress exposure affects thermal injury-induced mechanosensitivity in a female rat model of COSR. Adult female Sprague Dawley rats were exposed to the unpredictable combat stress (UPCS) procedure for either 2 or 4 weeks. The UPCS procedure included exposure to one stressor each day for four days. The stressors include: (1) sound stress for 30 min, (2) restraint stress for 4 h, (3) cold stress for 4 h, and (4) forced swim stress for 15 min. The order of stressors was randomized weekly. Mechanical and thermal sensitivity was tested twice weekly. After the UPCS procedure, a sub-set of rats received a thermal injury while under anesthesia. The development of mechanical allodynia and thermal hyperalgesia was examined for 14 days post-burn. UPCS exposure increased mechanosensitivity after two weeks. Interestingly, with more stress exposure, females seemed to habituate to the stress, causing the stress-induced changes in mechanosensitivity to decrease by week three of UPCS. If thermal injury induction occurred during peak stress-induced mechanosensitivity, after two weeks, this resulted in increased mechanical allodynia in the injured hind paw compared to thermal injury alone. This data indicates a susceptibility to increased nociceptive sensitization when injury is sustained at peak stress reactivity. Additionally, this data indicates a sex difference in the timing of peak stress. Post-mortem examination of the prefrontal cortex (PFC) showed altered expression of p-TrkB in 4-week stressed animals given a thermal injury, suggesting a compensatory mechanism. Future work will examine treatment options for preventing stress-induced pain to maintain the effectiveness and readiness of the Warfighter.

**Supplementary Information:**

The online version contains supplementary material available at 10.1186/s12868-022-00759-z.

## Introduction

Service Members experience various combat stressors on the battlefield, including both physical (e.g., extreme environments, illness, injury, etc.) and psychological stress (i.e., fear, anxiety, loss of a companion, etc.). Service Members exposed to battlefield stress experience lethality limiting symptoms such as fatigue, exhaustion, difficulty prioritizing tasks, headaches, back pain, anxiety, indecision, delayed reaction times, and poor concentration, they may be characterized as having a combat and operational stress reaction (COSR) [1, 2]. The term COSR describes the emergence of a broad range of maladaptive mental and behavioral symptoms that occur in response to battlefield stress. COSR differs from other acute stress disorders in that it is solely a military designation that is defined in terms of loss of functionality as a combatant and may not be linked to a specific traumatic event [3]. Further, some acute stress disorder symptoms, such as hyper startle and hypervigilance, would not be considered negative COSR symptoms as they may be beneficial during a combat situation [4, 5]. COSR also differs from chronic stress-related disorders, such as post-traumatic stress disorder (PTSD), in that COSR is the immediate reaction to combat stress exposure and is not persistent [4]. COSR is limited to individuals with symptoms during the first 72 h of onset. However, it is important to note that Soldiers that experience COSR are 6.6 times more likely to develop PTSD compared to Soldiers that do not experience COSR [6–8]. Current treatments for COSR include debriefing and initial rest and replenishment at medical combat stress control facilities followed by return to combat [2, 9].

Research has demonstrated that Service Members who experience COSR on the battlefield are at risk for somatization, lasting psychological problems, and poor physical health [7, 10–14]. In particular, one study found that COSR correlated with increased chest pains and headaches 20 years post-war [11]. In addition, research involving other diagnoses, such as PTSD, indicate lasting impairment in psychological and physical health when Soldiers sustain an injury on the battlefield [15]. Studies in civilian populations, which focus on the impact of stress or anxiety disorders on acute pain perception [16–18], also find that high anxiety scores correlate with higher pain scores. Additionally, a number of rodent models have shown that stress alone can increase pain sensitivity, a phenomena referred to as stress-induced hyperalgesia [19, 20]. However, few researchers have examined how exposure to acute stress immediately before an injury effects pain sensitization [21–27]. In addition, studies tend to use only one stressor, which can lead to contextualization of stress. Such models provide better representations of acute stress disorder rather than COSR, which tends to not be linked to a specific traumatic event. A recent study from our lab examined the effects of multiple stressors on pain outcomes in male rodents. This study found that prolonged exposure to unpredictable combat stressors (UPCS) caused stress-induced hyperalgesia. In addition, thermal injury to the rat hindpaw after UPCS exposure led to sustained mechanical allodynia compared to non-stressed injured controls [28]. Taken together, it appears that the effects of acute stress and pain may be additive. Thus, the combination of COSR and injury would inhibit Soldier lethality and mission accomplishment more than either one alone. Due to the nature of combat, it would be rare that either stress or trauma pain would occur independently. It is therefore of the utmost importance to research COSR effects on pain formation to elucidate a better understanding of this condition and thus discover best treatment options.

Although females have been historically restricted from combat roles, women do experience combat related injuries, especially in recent conflicts [29]. For example, between 2003 and 2014, there were 844 combat related injuries in females [30]. Data examining female Operation Enduring Freedom and Operation Iraqi Freedom service members sustaining combat-related injuries has shown that women may be as resilient to the effects of combat-related stress on post-deployment mental health as men. From 1973 to 2010, the number of active duty female Service Members expanded from 47,000 to over 167,000 [31]. It is expected that future conflicts will see this number grow, as women are now lawfully able to serve in combat zones. Currently, most of the literature (80%) examining the effects of stress or pain uses either human men or male rodents [32, 33]. Sexual dimorphism occurs early in development and can influence analgesia, drug potency, efficacy, and duration of action [34–38]. Research points to differences between males and females regarding response and treatment responsivity to pain, with females generally showing increased pain sensitivity [39]. However, effects can be inconsistent as differences in characterization of pain and models can greatly impact the results [39]. The effects of acute stress on pain sensitivity after injury in females is largely unknown. Given this, female-specific models are essential to test the efficacy and safety of analgesics and ensure proper pain management of all Service Members on the battlefield and at home. The present study therefore investigates how different amounts of prior stress exposure affects thermal injury-induced mechanosensitivity in a female rat model of combat stress exposure. This work aims to better understand how stress effects pain in both males and females to improve clinical management.

## Methods

### Animals

Sixty adult female Sprague–Dawley rats (seven to eight weeks old) were purchased from Charles River Laboratories, USA. Rats were quarantined for three days upon their arrival at the United States Army Institute of Surgical Research (USAISR) facility and then pair housed in cages with a 12 h light/dark cycle (6 am-6 pm) with access to food and water, ad libitum. Rats were acclimated to the vivarium for one week prior to experimental manipulations. Rats weighed 220–240 g at the start of the study and were 250–270 g at the conclusion of the study. Research was conducted in compliance with the Animal Welfare Act, the implementing Animal Welfare regulations, and the principles of the Guide for the Care and Use of Laboratory Animals, National Research Council. The facility’s Institutional Animal Care and Use Committee approved all research conducted in this study. The facility where this research was conducted is fully accredited by the AAALAC. Measures were taken to minimize the number of animals to be used for this study.

### Experimental design

**Fig. 1:**
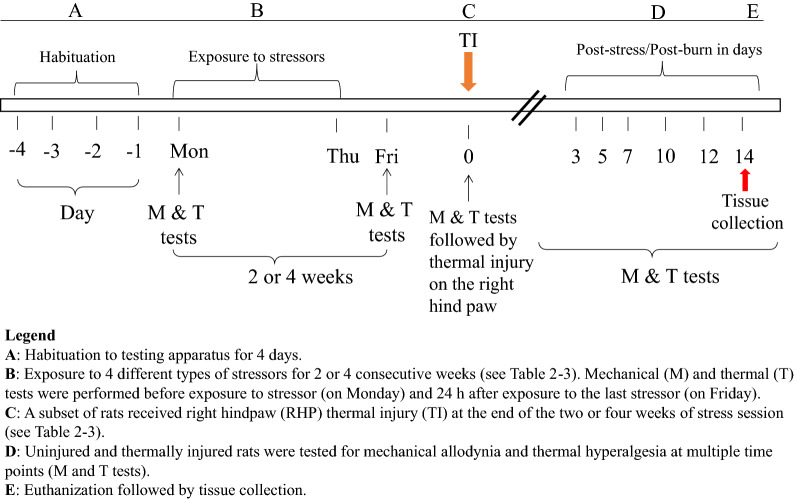
Timeline of experimental manipulations

A full factorial design was used to examine the effects of UPCS on nociceptive behaviors in uninjured and thermally injured rats, for a total of 4 groups: (1) NS- not stressed or injured, (2) S-stressed only, (3) NS + TI- thermal injury only, and (4) S + TI- stressed then thermal injured. All groups were habituated to handling and all experimental apparatuses for four days in the week prior (week 0) to starting the UPCS procedure (Fig. 1). During this habituation period, rats were exposed to each testing apparatus for 15 min/day. Beginning the Monday of week 1, all rats were tested for basal pain sensitivity to thermal and mechanical stimuli as described below. For rats receiving stress (Groups S and S + TI), the UPCS procedure was started following this sensitivity testing on the Monday of Week 1 and continued daily until Thursday, as shown in Fig. 1 and described below. S subjects were exposed to UPCS for either four (Experiment 1; n = 3) or two weeks (Experiment 2; n = 12). NS rats were exposed to the testing apparatus without the stressor as described below. Mechanical and thermal sensitivity testing was performed on Mondays and Fridays during the UPCS exposure period (Fig. 1B). After the 2 or 4-weeks of UPCS, half of the rats received a thermal injury while under anesthesia. All rats then had their thermal and mechanical sensitivity tested on days 3, 5, 7, 10, 12, and 14 post-injury (see Fig. 1D).

### UPCS procedure

Previous studies [40, 41], as well as our own preliminary experiments, have shown that each of the stressors that we employed in this study (forced swim stress (FSS), sound stress (SS), cold stress (CS), restraint stress (RS)) induce nociceptive behaviors. In order to avoid habituation of animals to a single type of predictable stressor and also to mimic clinical conditions, we exposed the animals to four different types of unpredictable stressors. Stressed rats were exposed to a different type of stressor (RS, FSS, SS, or CS) each day for four days (Monday–Thursday). Two days later, the same rats underwent behavioral testing (e.g., thermal and mechanical sensitivity testing) followed by exposure to a second stress session (rats were exposed to the same set of stressors but in a different order). This procedure was conducted for two or four consecutive weeks depending on the experiment. NS groups were handled, habituated to apparatus, and behaviors tested without being exposed to the stress procedures.

### Forced swim stress (FSS) protocol

Rats were placed in 30 cm depth of water in a round plastic container ~ 25 cm in diameter and 60 cm in height for 15 min at 25 ± 2 °C [42]. All rats were towel dried and returned to clean cages following each session. The plastic container was cleaned and new water was added for each subject. The NS rats were placed in the plastic container void of water for 15 min. After the stress session, rats were dried with towels and returned back to their home cages. Fecal pellets were collected from both the NS and S rats and weighed.

### Sound stress (SS) protocol

Rats were individually placed in an acrylic enclosure (8″ × 3 1⁄2″) contained in an acrylonitrile butadiene styrene (ABS) isolation chamber (Startle Response System apparatus, SR-Labs; San Diego Instruments, model numbers SIC002650-SIC002655) and exposed to 105 dB tone of mixed frequencies, ranging from 11 to 19 kHz each lasting for 5–10 s randomly each minute over a total of a 30 min period. This high frequency sound paradigm was chosen because (1) we have observed that 3 days of this paradigm results in nociceptive behavior [43] and (2) low frequency sound (~ 50 kB) results in elevated paw withdrawal threshold [44]. NS rats were placed in the acrylic enclosure within the ABS isolation chamber without the sound. Fecal pellets were collected from both the NS and S rats and weighed.

### Restraint stress (RS) protocol

Restraint stress was carried out by placing the animal in a restrainer (Harvard Apparatus: Cat#: 52-244 0494 for 250–500 g rat and 52-0486 for 125–250 g rat) for 4 h (from 9 am to 1 pm) without having access to food and water [40]. Rats were checked every half hour for overall health status. NS groups remained in their home cage with access to food and water. Fecal pellets were collected from both the NS and S rats and weighed.

### Cold stress (CS) protocol

To induce cold stress, rats in a new clean home cage were placed in a 4 °C cold room for 4 h with access to food and water. Rats were checked every hour for overall health status. NS groups were placed in new clean home cages at room temperature with access to food and water. Fecal pellets were collected from both the NS and S rats and weighed.

### Thermal injury (TI) induction protocol

Rats were anesthetized with isoflurane (3–4%) in oxygen and a slanted soldering tip connected to a temperature-controlled super soldering station was applied to the plantar surface of the right hindpaw for 30 s at a temperature of 100ºC. Wounds were treated with silver sulfadiazine (1%) ointment to prevent infection. No injury controls receive the same treatment except the soldering tip was at room temperature.

### Estrous cycle phase determination

Vaginal smears were collected to determine whether hormonal fluctuations impacted UPCS effect on mechanical testing or thermal injury induced nociceptive behavior. Female rats’ estrous cycle was monitored at the same time daily for 8–10 days prior to experimental manipulation to ensure that the rats were cycling normally. Every effort was made to ensure the same technician smeared the same animal set throughout the entire project for consistency in animal readings. Eyedroppers were filled with ~ 300 µl of deionized water, gently inserted into the animals vagina, and aspirated 2–3 times to collect cells. The sample was then immediately dispensed onto a concave microscope slide and assessed for the stage of estrous cycle using a light microscope. Four phases were used for categorization: (1) Diestrus 1 (leukocytes), (2) Diestrus 2 (leukocytes plus larger round cells without nuclei, (3) Proestrus (nucleated epithelial cells), (4) Estrus (cornified cells) [45]. Additional file 1: Table S1 indicates the number of female rats per category per time point measured.

### Detection of mechanical allodynia

An electric anesthesiometer (Ugo Basile) was used to assess paw withdrawal threshold (PWT) to a blunt mechanical stimulus as a measure of mechanical allodynia (increased sensitivity to a non-noxious stimulus). All rats were acclimated to the behavior testing room for 30 min and the testing apparatus for 20 min prior to behavioral testing. Rats were individually placed in clear plastic chambers (non-restrictive) on a grid and a blunt probe was applied to the plantar surface of the hindpaw in slowly increasing force until the animal voluntarily withdrew its paw. The grams of pressure required to elicit a paw withdrawal was recorded at multiple time points to gauge effects of experimental manipulations on allodynia. A smaller threshold indicates a greater sensitivity to a mechanical stimulus.

### Detection of thermal hyperalgesia

Testing of behavioral responses to heat was performed as described previously [46]. Rats were placed in Plexiglas chambers on a heated (30 °C) glass platform. A Paw Thermal Stimulator was used to stimulate the plantar hindpaw with a radiant light beam. The paw withdrawal latency (PWL) was automatically recorded by the apparatus. A maximal latency of 20-s was used to prevent tissue damage. Thermal hyperalgesia was measured in triplicate and the mean at each time point was analyzed. A smaller latency indicates a greater sensitivity to a thermal stimulus.

### Tissue isolation and microdissection

After the final behavioral experiments (Fig. 1 timeline), rats were humanely euthanized by decapitation and the brains were immediately removed, flash-frozen in liquid nitrogen and stored at − 80 °C until use. The whole intact frozen brain was placed in a pre-chilled rat brain slicing matrix (Zivic instruments) at 4 °C. The brain was maintained in a semi-frozen state and all dissections were completed prior to thawing. To obtain the hypothalamic region, a coronal section was taken from − 0.40 mm to − 4.30 mm from bregma [47]. The left and right hemisphere was separated at the corpus callosum and four mm thick punches from the left and right hypothalamic region were taken [48]. For dissection of the frontal cortex we followed the dissection parameters as shown earlier [49]. The frontal cortex was separated from the whole brain by cutting at the first appearance of the corpus callosum at bregma 0.70 mm [28, 49]. The ventral area containing the olfactory nuclei was removed; leaving the dorsal prefrontal cortex intact [28, 49]. This was further separated into left and right hemispheric regions.

### Protein isolation

To isolate protein, 4-(2-Hydroxyethyl) piperazine-1-ethanesulfonic acid (HEPES) based (20 mM HEPES; 1 mM EDTA; 40 units/mL RNAse inhibitor; mini complete protease inhibitor tablet) buffer was added to the dissected right (ipsilateral) and left (contralateral) dorsal prefrontal cortex and hypothalamic samples. Tissue was homogenized twice for 20 s, split into two separate tubes, and centrifuged at 14000xg for 20 min at 4 °C. Tri-reagent was added to the supernatant of one set of tubes followed by RNA isolation using the Zymogen Directzol RNA miniprep kit (ZRC175939). RNA concentration was determined by Nanodrop instrument. The pellet for the protein isolate was solubilized in radioimmunoprecipitation assay (RIPA) buffer (with protease and phosphatase inhibitors) for 20 min on ice. Following another centrifugation step, the supernatant was subjected to bicinchoninic acid (BCA; Pierce) assay to determine protein concentration.

### Simple Wes

Glycosylated TrkB (LSBio, cat#: LS-C48549) and phosphorylated TrkB (LSBio, cat#: LS-C95153) and total protein expression was determined by Wes™ analysis (ProteinSimple cat#: SM-W004 (12–230 kDa Separation Module), DM-001 (Anti-rabbit detection module), DM-TP01 (Total protein detection module)) following the manufacturer’s directions [28]. The 5X fluorescent master mix was prepared with 400 mM dithiothreitol (DTT) and 10X sample buffer. The biotinylated ladder was prepared with 10X sample buffer, 400 mM DTT, and deionized water, denatured for five minutes at 95 °C, and loaded into lane one of the pre-filled plate provided by protein simple [28]. The prepared 5X fluorescent master mix was combined with lysate for a final protein concentration of 0.2 mg/ml. The TrkB primary antibody (1:50 dilution) and luminol-S/peroxide combined substrate was prepared and loaded onto the plate following the assay plate layout designed by protein simple [28]. Data analysis was conducted utilizing the Wes™ and ImageJ software.

### Hindpaw sectioning and H&E staining

Tissue specimens were placed in formalin, then decalcified with Immunocal® for 21 days, processed by conventional methods, embedded in paraffin, sectioned to four micrometers, and stained with hematoxylin and eosin. Slides were examined and scored by a board certified veterinary pathologist as shown in Table 1.

**Table 1:** Scoring of H&E stained burn injured hind paws

Epidermis	
0	Normal
1	Epidermal degeneration
2	Epidermal ulceration
3	Epidermal hyperplasia
Granulation tissue/Fibroplasia	
0	None
1	GT > Fibroplasia
2	GT = Fibroplasia
3	GT < Fibroplasia
Skeletal myocyte degeneration, necrosis, atrophy, loss	
0	Normal
1	< 25% (% of total skeletal muscle)
2	26–50% (% of total skeletal muscle)
3	51–75% (% of total skeletal muscle)
4	> 75% (% of total skeletal muscle)
Superficial bacteria	
0	Absent
1	Present
Inflammation (neutrophils/histiocytes/lymphocytes and plasma cells)	
0	None
1	Mild number of inflammatory cells
2	Moderate number of inflammatory cells
3	Marked number of inflammatory cells
New bone growth (digit)	
0	None
1	Mild
2	Moderate
3	Marked
4	Severe

Slides were examined and scored by a board certified veterinary pathologist utilizing the scoring system detailed in Table 1

### Statistical analysis

GraphPad and jamovi were used to perform all statistical tests. Significance was set at 0.05. Post hoc comparisons were conducted using Bonferroni Post hoc multiple comparison test. All experiments were performed in a blinded fashion. For uninjured rats, we combined the left and right hind paws PWT or PWL for analysis.

## Results

### Effects of four weeks of UPCS exposure on thermal injury-induced outcomes

Previous research has shown that four weeks of stress can cause changes in mechanosensivity in males [28]. Additionally, animals given an injury (i.e., burn to the paw) after stress exposure showed a further increase in mechanosensivity compared to those given an injury alone [28]. To explore whether a similar effect is seen in females, we exposed female rats to four weeks of UPCS (Fig. 1). Table 2 indicates which stress exposure was performed each day. After the four weeks, half of the animals received a thermal injury to the paw. Mechanosensitivity was examined throughout the experimental procedure.

**Table 2:** Stress exposure during weeks 1–4

Week	Day	Stressor
1	Monday	Restraint stress (RS)
1	Tuesday	Forced swim stress (FSS)
1	Wednesday	Sound stress (SS)
1	Thursday	Cold stress (CS)
2	Monday	FSS
2	Tuesday	SS
2	Wednesday	CS
2	Thursday	RS
3	Monday	CS
3	Tuesday	SS
3	Wednesday	RS
3	Thursday	FSS
4	Monday	SS
4	Tuesday	RS
4	Wednesday	FSS
4	Thursday	CS

Stress exposure each day per week for 4 weeks is detailed in Table 2

### Four weeks of UPCS exposure caused an increase in mechanosensitivity in females

**Fig. 2:**
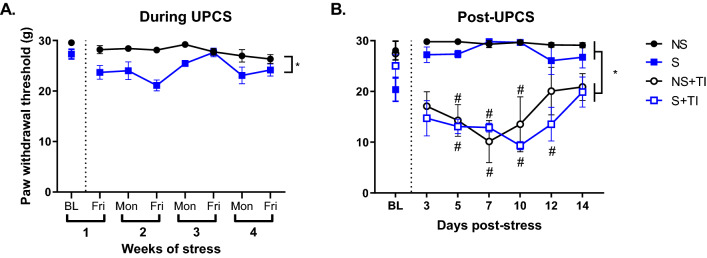
Mechanical allodynia during and after four weeks of UPCS exposure. Stress decreased PWT in female rats compared to controls (**A**). Thermal injury caused a decrease in PWT in both S + TI and NS + TI groups (**B**). Asterisks indicate significant difference between groups, while hashtags indicate differences from baseline, p < 0.05. Error bars represent SEM (n = 3–6)

Using a two-way ANOVA with stress as the dependent measure and time as a repeated measure, we found a main effect of Stress and Time, *F* (1, 10) = 79.2, *p* < 0.001 and *F* (7, 70) = 3.56, *p* = 0.003, respectively. No other effects were statistically significant, *F* (7, 70) = 1.94, *p* = 0.077) (Fig. 2a). Post hoc analysis found that animals in the S group had a lower PWT compared to NS controls. No other groups were statistically significant, *p* > 0.05.

### Thermal injury caused an increase in mechanosensitivity in females but was not impacted by four weeks of prior UPCS exposure

Using a three-way ANOVA with stress and injury as dependent measures and time as a repeated measure, we found a main effect of Time and Injury, *F* (6, 48) = 2.382, *p* = 0.043 and *F* (1, 8) = 137.6247, *p* < 0.001, respectively (Fig. 2b). There was also an interaction between Time x Injury and Stress x Injury, *F* (6, 48) = 9.147, *p* < 0.001 and *F* (1, 8) = 5.7004, *p* = 0.044, respectively. No other effects were statistically significant, all *F*s < 1.0, *p* > 0.5. Post hoc analysis of the main effects found that the TI groups showed lower PWT compared to uninjured animals. Analysis of the interactions found that TI resulted in significant reduction in PWT at time points 5–10 and 5–12 days post-stress for NS + TI and S + TI, respectively, compared to baseline. Analysis of the stress by injury interaction showed that subjects in the NS group differed significantly from subjects in the NS + TI group. No other groups were statistically significant, *p* > 0.05.

### TrkB signaling in the hypothalamus, but not in the PFC, was increased in rats receiving 4 weeks of UPCS and thermal injury

**Fig. 3:**
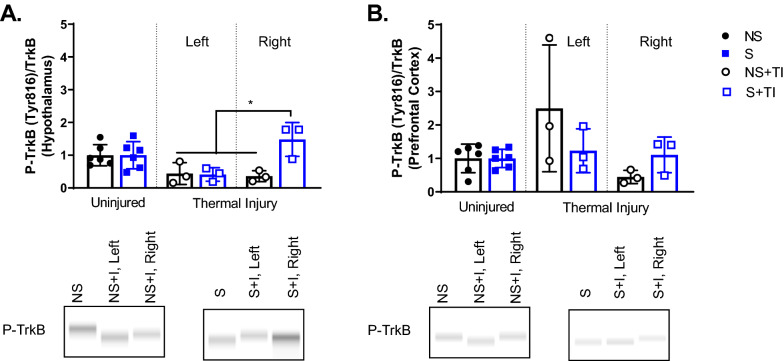
Changes in TrkB signaling in the hypothalamus and prefrontal cortex after four weeks of UPCS. TrkB signaling in the hypothalamus was increased in the S + TI subjects showing the highest expression on the right side compared to the other injured groups (**A**). There was no difference in TrkB signaling in the PFC (**B**). Asterisks indicate significant difference between groups, p < 0.05. Error bars represent SEM (n = 3–6)

An ANOVA examining TrkB levels in the hypothalamus found a main effect of Side and Stress and an interaction between Side x Stress, *F* (2, 18) = 5.45, *p* = 0.014, *F* (1, 18) = 5.69, *p* = 0.028, and *F* (2, 18) = 5.69, *p* = 0.012, respectively (Fig. 3a). Post hoc analysis of the main effects found higher TrkB signaling in the hypothalamus of S + TI subjects and lower levels of TrkB on the left side compared to the right side and controls. Analysis of the interaction showed that S + TI on the right side was significantly different then left S + TI and NS groups.

An ANOVA examining TrkB levels in the PFC found a main effect of side, *F* (2, 18) = 3.757, *p* = 0.043. No other effects were statistically significant, all Fs < 1.00, *p* > 0.09. While post hoc analysis did not reveal any significant differences between groups, the left side generally had more TrkB signaling compared to the right side and controls, *p* = 0.052 (Fig. 3b).

### Pathology report

We hypothesized that UPCS exposure may affect wound healing and therefore performed H&E staining and analysis. However, there were no significant differences between S + TI and NS + TI animals on the pathology measurements examined, Mann–Whitney *U* > 1.5, *p* > 0.197 (Fig. 8, Additional file 1: : Fig. S2).

## Effects of two weeks of UPCS exposure on thermal injury-induced outcomes

In the previous experiment, we saw that four weeks of stress caused changes in mechanosensitivity, but failed to effect sensitivity after thermal injury. This was unexpected given previous research in male rats. We hypothesized that this difference could be due to the difference in mechanosensitivity over the four weeks of UPCS in females. While males show high levels of mechanosensitivity throughout the four weeks of UPCS, this effect seems to peak around two weeks of UPCS for female rats (Fig. 2a). Because of these findings, we decided to repeat this experiment but thermally injure the rats at two weeks of UPCS exposure, when the nociceptive behavior is at maximum. The experimental paradigm and order of stressor are indicated in Fig. 1 and Table 3.

**Table 3:** Stress exposure during weeks 1–2

Week	Day	Stressor
1	Monday	Restraint stress (RS)
1	Tuesday	Forced swim stress (FSS)
1	Wednesday	Sound stress (SS)
1	Thursday	Cold stress (CS)
2	Monday	FSS
2	Tuesday	SS
2	Wednesday	CS
2	Thursday	RS

Stress exposure each day per week for 2 weeks is detailed in Table 3

### Two weeks of UPCS exposure increased mechanosensitivity in females

**Fig. 4:**
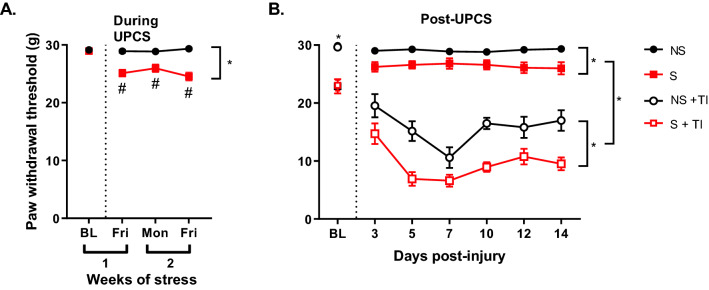
Mechanical allodynia during (Fig. 4a) and after (Fig. 4b) two weeks of UPCS. Asterisks indicate significant difference between groups, while hashtags indicate differences from baseline, p < 0.05. Error bars represent SEM (n = 12–18)

A two-way ANOVA with stress as the dependent variable and time as a repeated measure found a main effect of Time and Stress, and an interaction between Time x Stress, *F* (3, 84) = 12.0, *p* < 0.001, *F* (1, 28) = 77.5, *p* < 0.001, and *F* (3, 84) = 12.1, *p* < 0.001, respectively (Fig. 4a). Post hoc analysis of the main effects showed that S subjects had lower PWT compared to NS subjects. Analysis of the interaction showed that S subjects showed lower PWT compared to their baseline.

### Thermal injury caused an increase in mechanosensitivity in females and this was further increased after 2 weeks of UPCS exposure

The first two weeks of stress exposure resulted in a significant reduction in PWT compared to the NS group (Fig. 4a). A three-way ANOVA with stress and injury as the dependent variable and time as a repeated measure found a main effect of Time, Injury and Stress, *F* (6, 156) = 21.72, *p* < 0.001, *F* (1, 26) = 187.34, *p* < 0.001, *F* (1,26) = 24.48, *p* < 0.001, respectively (Fig. 4b). NS + TI and S + TI groups were significantly different. No other effects were statistically significant, all *F*s < 1.0, *p* > 0.06. Post hoc analysis of the main effects showed that thermal injury resulted in a significant reduction of PWT compared to uninjured controls. Further, subjects pre-exposed to stress showed lower PWT compared to non-stressed subjects even after stress exposure was complete (Fig. 4b).

### Two weeks of UPCS exposure did not alter TrkB signaling in the hypothalamus and PFC

**Fig. 5:**
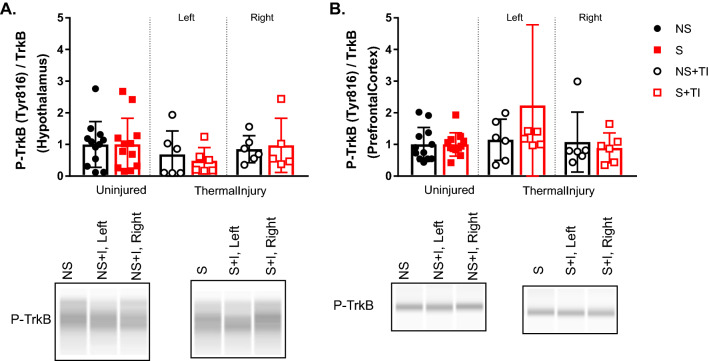
Changes in TrkB signaling in the hypothalamus and prefrontal cortex after two weeks of UPCS. TrkB signaling was not different in the hypothalamus (**A**) and PFC (**B**) of stressed and injured female rats after two weeks of UPCS. Asterisks indicate significant difference between groups, p < 0.05. Error bars represent SEM (n = 6–12)

An ANOVA revealed no significant differences in TrkB signaling in the hypothalamus and PFC of stressed or injured animals, all *F*s < 1.0, *p* > 0.1 (Fig. 5a, b).

### Two weeks of UPCS exposure did not increase thermal sensitivity

**Fig. 6:**
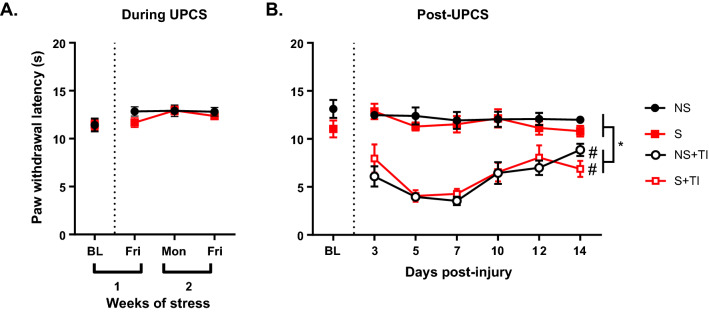
Thermal sensitivity during and after two weeks of UPCS. Two weeks of UPCS did not change PWL (**A**). Thermal injury caused a significant decrease in PWL compared to uninjured and BL values (**B**). Asterisks indicate significant difference between groups, while hashtags indicate differences from baseline, p < 0.05. Error bars represent SEM (n = 12–18)

An ANOVA with stress as the dependent variable and time as the repeated measure revealed a main effect of Time, *F* (3, 102) = 5.31, *p* = 0.002 (Fig. 6a). No other effects were statistically significant, all *F*s < 1.0, *p* > 0.1. Post hoc analysis of the main effect found that thermal sensitivity was different from baseline during week two.

### Thermal injury increased thermal sensitivity, but there was no effect of stress

Using a three-way ANOVA with stress and injury as the dependent variables and time as a repeated measure, we found a main effect of Time, Stress, and Injury, and an interaction between Time x Injury, *F* (6, 120) = 16.227, *p* < 0.001, *F* (1, 20) = 4.618, *p* = 0.044, *F* (1, 20) = 168.574, *p* < 0.001 and *F* (6, 120) = 11.695, *p* < 0.001, respectively (Fig. 6b). No other effects were statistically significant, all *F*s < 1.0, *p* > 0.4. Post hoc analysis of the main effects found that thermal injury decreased PWL in NS + TI and S + TI groups. Analysis of the interaction found that PWL was different from BL for injured groups from days 3–14 post-stress.

### Two weeks of UPCS exposure increased fecal pellet weight, but had no effect on weight gain

**Fig. 7:**
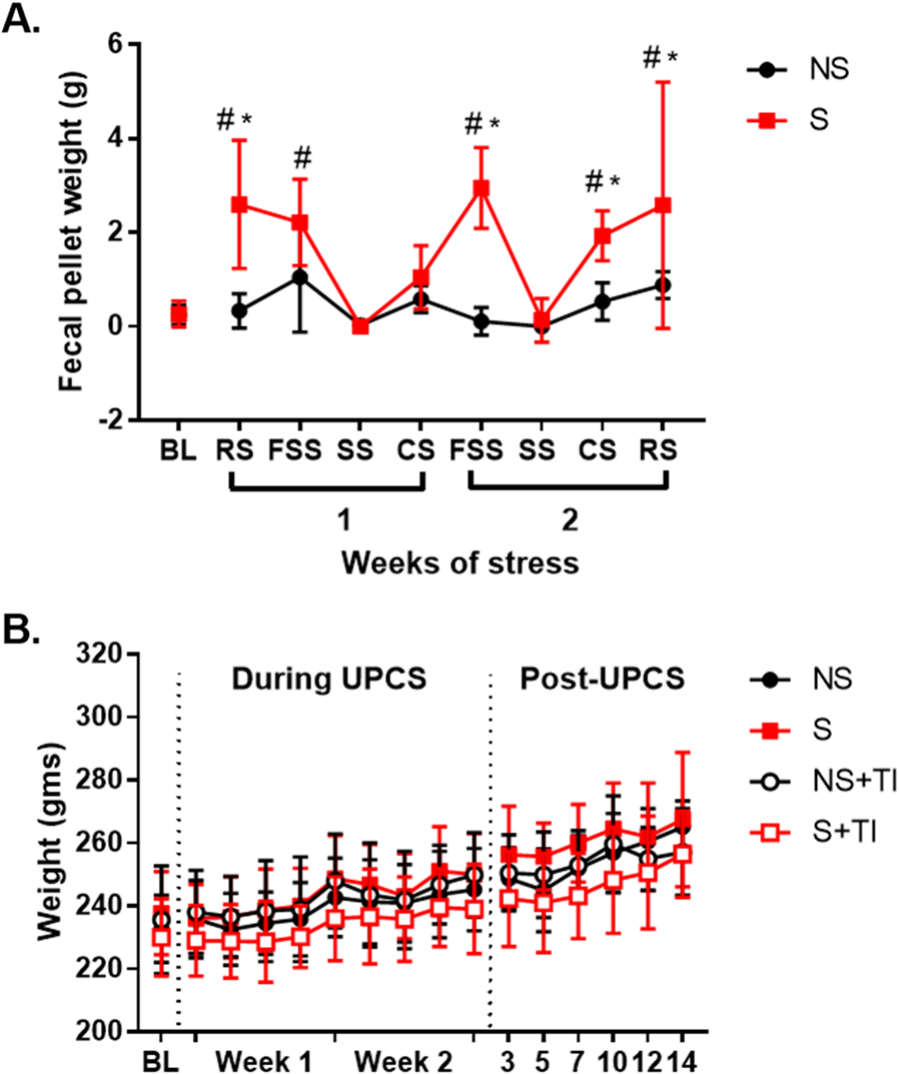
Changes in animal body weight and fecal pellets during two week of UPCS. Fecal pellet weight was increased in stressed rats, especially during the second week of UPCS exposure compared to controls and baseline measures (**A**). Weight increased over the course of the experiment in all subjects (**B**). Asterisks indicate significant difference between groups, while hashtags indicate differences from baseline, p < 0.05. Error bars represent SEM (n = 12)

Fecal pellet weight was measured during the two weeks of UPCS exposure (Fig. 7a). Overall analysis using an ANOVA with stress as the dependent variable and time as the repeated measure, found a main effect of Time and Stress and an interaction between Time x Stress, *F* (8, 176), *p* < 0.001, *F* (1, 22), *p* < 0.001 and *F* (8, 176), *p* < 0.001. Post hoc analysis of the main effect showed that animals exposed to stress (S) had higher fecal pellet weight. Analysis of the interaction showed a significant increase in fecal pellet weight for RS and FSS during week 1 and FSS, CS, and RS during week two in S subjects compared to baselines. Further, S differed from NS at RS week one and FSS, CS and RS week two. No other groups were statistically significant, *p* > 0.05.

Examination of the effect of stress on weight before injury using a two-way ANOVA with time as the repeated measure found a main effect of Time, *F* (9, 414) = 52.97, *p* < 0.001 (Fig. 7b). No other effects were statistically significant, all Fs < 1.0, p > 0.3. To examine whether injury and stress affected weight, we used a three-way ANOVA with stress and injury as dependent variables and time of a repeated measure. Results indicated a main effect of Time, *F* (6, 120) = 49.402, *p* < 0.001. No other effects were statistically significant, all Fs < 4.0, p > 0.086. Post hoc analysis of the main effect showed that body weight steadily increased for all groups over time.

### Pathology report

**Fig. 8:**
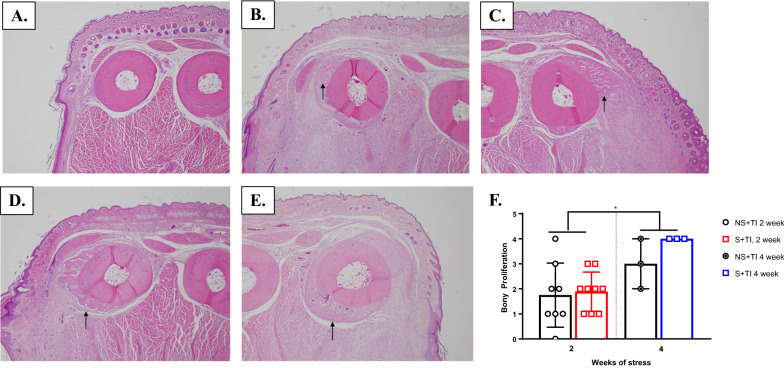
Bony Proliferation scores after two and four weeks of UPCS. There were no significant differences between S + TI and NS + TI animals on the different pathology measurements examined. However, we further examined whether there were changes in pathology depending on the two stress time groups (NS + TI and S + TI, 2 weeks UPCS or NS + TI and S + TI, 4 weeks UPCS) (Fig. 8a-f). There was a significant difference between these two groups on bony proliferation, with the NS + TI and S + TI, 4 weeks showing more bone formation compared to the NS + TI and S + TI, 2 weeks (Fig. 8a-f). Additionally, while not significant, 4 weeks of UPCS exposure prior to TI showed elevated bony proliferation compared to NS + TI, 4 weeks (Fig. 8a-f). B-E: Black arrow is pointing to the bony proliferation observed in the S + TI, 4-week UPCS group (Fig. 8a-f). Asterisks indicate significant difference between groups, p < 0.05. Error bars represent SEM (n = 3-9)

There were no significant differences between S + TI and NS + TI animals on the different pathology measurements examined, Mann–Whitney *U* > 26.5, *p* > 0.550 (Fig. 8, Additional file 1: : Fig. S3). We also examined whether there were changes in pathology depending on the 2 stress time groups (NS + TI and S + TI, 2 weeks UPCS or NS + TI and S + TI, 4 weeks UPCS). We found a significant difference between these 2 groups on bony proliferation, with the NS + TI and S + TI, 4 weeks showing more bone formation (Man Whitney *U* = 11.5, *p* = 0.005) compared to the NS + TI and S + TI, 2 weeks. Additionally, while not significant, 4 weeks of UPCS exposure prior to TI showed elevated bony proliferation compared to NS + TI, 4 weeks (Fig. 8). No other pathology measurements were statistically different (Mann Whitney *U* > 31.5, *p* > 0.182.

## Discussion

The results from this study demonstrate that the duration of UPCS exposure has a significant impact on the mechanosensitivity experienced by female rats. Maximum mechanosensitivity is observed at two weeks of UPCS exposure with female rats recovering to baseline levels by the end of Week 3. When thermal injury was induced at four weeks of UPCS, when no effect of UPCS on basal mechanosensitivity was observed, no differences in thermal injury induced mechanosensitivity was seen. However, when thermal injury was induced at two weeks of UPCS exposure, stress caused an increase in mechanosensitivity.

One major difference between males and females is the timeline of changes in basal mechanosensitivity that results from UPCS exposure. When we compare this data with our previously published male data using the same UPCS model, we can see differences between the sexes [28]. The duration of the UPCS exposure needed to induce changes in mechanosensitivity is different between males and females. In males, it takes longer to see an effect of stress, whereas females react quickly to UPCS and then recover to baseline levels.

This study indicates that there are differences in UPCS and UPCS-exacerbated thermal injury induced mechanosensitivity between male and female rats. There are also some similarities between male and female rats in terms of their responses to thermal stimulus. For both sexes, PWL was reduced following thermal injury when compared to BL but no significant difference was observed between the NS + TI and S + TI groups.

Furthermore, there are differences in molecular mechanisms mediating UPCS and UPCS-exacerbated thermal injury induced mechanosensitivity between male and female rats. The results from this study indicate that hypothalamic TrkB signaling may play a compensatory role following 4 weeks of UPCS exposure and thermal injury in female rats. P-TrkB levels were upregulated in the right (ipsilateral) side of the hypothalamus in the S + TI group when compared to the left (contralateral) side of the hypothalamus in the S + TI group (Fig. 3a). This was unexpected due to the decussation of nerve fibers within the spinal cord. We hypothesized that most of the molecular changes would occur on the left side of the brain as the TI was on the right hindpaw. This led us to hypothesize that these changes in the right side are compensatory mechanisms that may explain why the mechanosensitivity recovers to BL levels by 4 weeks of UPCS. Furthermore, when thermal injury induced mechanosensitivity is observed at 2 weeks of UPCS, no changes in TrkB signaling is observed (Fig. 5a and b). This is significantly different from the previously published male results. In our previous findings, we found that stressed male rats with thermal injury had an augmented level of TrkB and p-TrkB in the hypothalamus.

While the order of stressors for the first two weeks of stress exposure is the same for males and females, one limitation of the study is that on the day immediately prior to thermal injury in the 2 week UPCS group the female rats are exposed to restraint stress whereas in the 4 week UPCS group the male rats are exposed to cold stress. This may explain some of the differences observed in TrkB signaling. Current unpublished RNA sequencing studies performed by our lab show gene expression changes can occur in both the contralateral and ipsilateral side of the brain. Additionally, observing TrkB changes in the ipsilateral side of the hypothalamus, while somewhat unexpected, could be potentially explained by the combination of the UPCS exposure and thermal injury. UPCS effects would not be confined to the contralateral side of the brain. BDNF-TrkB signaling has been observed to contribute to pain-induced anhedonia where tissue levels of BDNF in the nucleus accumbens are elevated in comparison to control rats and rats without anhedonia-like phenotype [50]. A separate study indicates that in the oval nucleus of the bed nucleus of the stria terminalis (ovBNST), BDNF-TrkB signaling functions to dampen excitability to potentially mediate ovBNST role of reducing maladaptive behaviors associated with stress [51]. A similar mechanism could be occurring in the hypothalamus. Additionally, activation of BDNF-TrkB signaling is one mechanism of action for conventional and fast-acting antidepressants [52].

In conclusion, there are significant differences and similarities in UPCS exposed and thermal injured male and female rats. Substantial alterations in behavioral and molecular outcomes are observed between the two sexes. UPCS exposure results in increased basal mechanosensitivity that further exacerbates thermal injury-induced nociceptive behavior. This study highlights the notion that sex may play an important role in pain sensitivity. As precision medicine advances, identifying sex-based differences related to pain may help guide treatment decisions in the future. Understanding that sex potentially influences treatment or the timing of treatment has the potential to optimize healthcare. Studies using models that evaluate both male and female responses to stress, pain, injury, and/or treatment efficacy are therefore of the utmost importance to both military medicine and the overall scientific community.

## Supplementary Information

**Additional file 1.** Raw blots were provided for Figures 3a, 3b, 5a, and 5b. Raw data was provided for Figures 2a, 2b, 4a, 4b, 6a, 6b,7a, and 7b.

## Data Availability

All data generated or analyzed during this study are included in this published article [and its supplementary information files].

## References

[1] Solomon Z (2019). Is acute stress reaction a risk factor for early mortality?. Health Psychol.

[2] Solomon Z, Shklar R, Mikulincer M (2005). Frontline treatment of combat stress reaction: a 20-year longitudinal evaluation study. Am J Psychiatry.

[3] Cardena E, Carlson E (2011). Acute stress disorder revisited. Annu Rev Clin Psychol.

[4] Brewin CR (2017). A review of current evidence regarding the ICD-11 proposals for diagnosing PTSD and complex PTSD. Clin Psychol Rev.

[5] Davidson JR, Foa EB (1991). Diagnostic issues in posttraumatic stress disorder: considerations for the DSM-IV. J Abnorm Psychol.

[6] Solomon Z (1994). PTSD among Israeli former prisoners of war and soldiers with combat stress reaction: a longitudinal study. Am J Psychiatry.

[7] Solomon Z (2006). Reactions to combat stress in Israeli veterans twenty years after the 1982 Lebanon war. J Nerv Ment Dis.

[8] Isserlin L, Zerach G, Solomon Z (2008). Acute stress responses: a review and synthesis of ASD, ASR, and CSR. Am J Orthopsychiatry.

[9] True PK, Benway MW (1992). Treatment of stress reaction prior to combat using the "BICEPS" model. Mil Med.

[10] Solomon Z (1993). Combat stress reaction: the enduring toll of war.

[11] Benyamini Y, Solomon Z (2005). Combat stress reactions, posttraumatic stress disorder, cumulative life stress, and physical health among Israeli veterans twenty years after exposure to combat. Soc Sci Med.

[12] Stratton KJ (2014). Longitudinal interactions of pain and posttraumatic stress disorder symptoms in U.S. Military service members following blast exposure. J Pain.

[13] Giordano NA (2018). Complexity of the relationships of pain, posttraumatic stress, and depression in combat-injured populations: an integrative review to inform evidence-based practice. Worldviews Evid Based Nurs.

[14] Chapman PL (2012). Training, deployment preparation, and combat experiences of deployed health care personnel: key findings from deployed U.S. Army combat medics assigned to line units. Mil Med.

[15] Flandreau EI, Toth M (2018). Animal models of PTSD: a critical review. Curr Top Behav Neurosci.

[16] Taenzer P, Melzack R, Jeans ME (1986). Influence of psychological factors on postoperative pain, mood and analgesic requirements. Pain.

[17] Carr EC, NickyThomas V, Wilson-Barnet J (2005). Patient experiences of anxiety, depression and acute pain after surgery: a longitudinal perspective. Int J Nurs Stud.

[18] Boeke S (1991). Prediction of postoperative pain and duration of hospitalization using two anxiety measures. Pain.

[19] Michaelides A, Zis P (2019). Depression, anxiety and acute pain: links and management challenges. Postgrad Med.

[20] Jennings EM (2014). Stress-induced hyperalgesia. Prog Neurobiol.

[21] Wang PK (2015). Short-term sleep disturbance-induced stress does not affect basal pain perception, but does delay postsurgical pain recovery. J Pain.

[22] Robbins MT, Ness TJ (2008). Footshock-induced urinary bladder hypersensitivity: role of spinal corticotropin-releasing factor receptors. J Pain.

[23] Cao J (2015). Short-term pre- and post-operative stress prolongs incision-induced pain hypersensitivity without changing basal pain perception. Mol Pain.

[24] Asmundson GJ, Katz J (2009). Understanding the co-occurrence of anxiety disorders and chronic pain: state-of-the-art. Depress Anxiety.

[25] Wiech K, Tracey I (2009). The influence of negative emotions on pain: behavioral effects and neural mechanisms. Neuroimage.

[26] Zhang Z (2019). The GCs-SGK1-ATP signaling pathway in spinal astrocytes underlied presurgical anxiety-induced postsurgical hyperalgesia. Anesth Analg.

[27] Liu Y (2015). The activation of spinal astrocytes contributes to preoperative anxiety-induced persistent post-operative pain in a rat model of incisional pain. Eur J Pain.

[28] Sosanya NM (2019). Involvement of brain-derived neurotrophic factor (BDNF) in chronic intermittent stress-induced enhanced mechanical allodynia in a rat model of burn pain. BMC Neurosci.

[29] Street AE, Vogt D, Dutra L (2009). A new generation of women veterans: stressors faced by women deployed to Iraq and Afghanistan. Clin Psychol Rev.

[30] Dye JL (2016). Characterization and comparison of combat-related injuries in women during OIF and OEF. Mil Med.

[31] Patten E, Parker K (2011). Women in the US military: growing share, distinctive profile.

[32] Loyd DR, Murphy AZ (2014). The neuroanatomy of sexual dimorphism in opioid analgesia. Exp Neurol.

[33] Mogil JS (2020). Qualitative sex differences in pain processing: emerging evidence of a biased literature. Nat Rev Neurosci.

[34] Greenspan JD (2007). Studying sex and gender differences in pain and analgesia: a consensus report. Pain.

[35] Mogil JS, Bailey AL (2010). Sex and gender differences in pain and analgesia. Prog Brain Res.

[36] Nasser SA, Afify EA (2019). Sex differences in pain and opioid mediated antinociception: modulatory role of gonadal hormones. Life Sci.

[37] Long CC, Sadler KE, Kolber BJ (2016). Hormonal and molecular effects of restraint stress on formalin-induced pain-like behavior in male and female mice. Physiol Behav.

[38] Vogt D (2011). Gender differences in combat-related stressors and their association with postdeployment mental health in a nationally representative sample of U.S. OEF/OIF veterans. J Abnorm Psychol.

[39] Bartley EJ, Fillingim RB (2013). Sex differences in pain: a brief review of clinical and experimental findings. Br J Anaesth.

[40] Gamaro GD (1998). The effects of acute and repeated restraint stress on the nociceptive response in rats. Physiol Behav.

[41] Khasar SG, Green PG, Levine JD (2005). Repeated sound stress enhances inflammatory pain in the rat. Pain.

[42] Nyland JE, McLean SA, Averitt DL (2015). Prior stress exposure increases pain behaviors in a rat model of full thickness thermal injury. Burns.

[43] Sosanya N (2017). Sound-stress-induced altered nociceptive behaviors are associated with increased spinal CRFR2 gene expression in a rat model of burn injury. J Pain Res.

[44] Zhou W (2022). Sound induces analgesia through corticothalamic circuits. Science.

[45] Becker JB (2005). Strategies and methods for research on sex differences in brain and behavior. Endocrinology.

[46] Fowler M (2014). A rat model of full thickness thermal injury characterized by thermal hyperalgesia, mechanical allodynia, pronociceptive peptide release and tramadol analgesia. Burns.

[47] Rivera HM (2012). Estradiol increases the anorexia associated with increased 5-HT(2C) receptor activation in ovariectomized rats. Physiol Behav.

[48] Figini M (2015). In vivo DTI tractography of the rat brain: an atlas of the main tracts in Paxinos space with histological comparison. Magn Reson Imaging.

[49] Schulte-Herbruggen O (2006). Stress-resistant mice overexpressing glucocorticoid receptors display enhanced BDNF in the amygdala and hippocampus with unchanged NGF and serotonergic function. Psychoneuroendocrinology.

[50] Fang X (2020). Brain-derived neurotrophic factor-TrkB signaling in the medial prefrontal cortex plays a role in the anhedonia-like phenotype after spared nerve injury. Eur Arch Psychiatry Clin Neurosci.

[51] Fiedler D (2021). Brain-derived neurotrophic factor/tropomyosin receptor kinase B signaling controls excitability and long-term depression in oval nucleus of the BNST. J Neurosci.

[52] Vakhitova YV (2021). Analysis of antidepressant-like effects and action mechanisms of GSB-106, a small molecule, affecting the TrkB signaling. Int Jour Mol Sci.

